# Characterization of *Staphylococcus aureus* isolates from faecal samples of the Straw-Coloured Fruit Bat (*Eidolon helvum*) in Obafemi Awolowo University (OAU), Nigeria

**DOI:** 10.1186/1471-2180-12-279

**Published:** 2012-11-26

**Authors:** Babatunji Akobi, Oladipo Aboderin, Takashi Sasaki, Adebayo Shittu

**Affiliations:** 1 Department of Microbiology, Obafemi Awolowo University, Ile-Ife, Nigeria; 2 Department of Medical Microbiology and Parasitology, Obafemi Awolowo University, Ile-Ife, Nigeria; 3 Laboratory of Bacterial Genomics, Pathogen Genomics Center, National Institute of Infectious Diseases, Tokyo, Japan

**Keywords:** *Staphylococcus aureus*, *Eidolon helvum*, ST1725, ST1726, ST2463, ST2470, Anciently-diverged *S. aureus*

## Abstract

**Background:**

Bats (*Chiroptera*) are one of the most diverse groups of mammals which carry out important ecological and agricultural functions that are beneficial to humans. However, they are increasingly recognized as natural vectors for a number of zoonotic pathogens and favourable hosts for zoonotic infections. Large populations of the Straw-Coloured Fruit Bat (*Eidolon helvum*) colonize the main campus of the Obafemi Awolowo University (OAU), Ile-Ife, Nigeria, but the public health implications of faecal contamination and pollution by these flying mammals is unknown. This study characterized *S. aureus* obtained from faecal samples of these migratory mammals with a view to determining the clonal types of the isolates, and to investigate the possibility of these flying animals as potential reservoir for zoonotic *S. aureus* infections.

**Results:**

One hundred and seven (107) *S. aureus* isolates were recovered from 560 faecal samples in eleven roosting sites from January 2008 to February 2010. A large proportion of the isolates were susceptible to antibiotics, and molecular characterization of 70 isolates showed that 65 (92.9%) were assigned in coagulase type VI, while accessory gene typing classified 69 isolates into the following: type I (12; 17.1%), type II (3; 4.3%), type III (1; 1.4%) and type IV (53; 75.7%). On the whole, the isolates were grouped in five (A-E) main genotypes. Of the ten representative isolates selected for multilocus sequence typing (MLST), nine isolates were assigned with new sequence types: ST1725, ST1726, ST1727, ST2463-ST2467 and ST2470. Phylogenetic analysis provided evidence that *S. aureus* isolates in group C were closely related with ST1822 and associated clones identified in African monkeys, and group D isolates with ST75, ST883 and ST1223. The two groups exhibited remarkable genetic diversity compared to the major *S. aureus* clade.

**Conclusions:**

Antibiotic resistance in faecal *S. aureus* isolates of *E. helvum* is low and multiple unique *S. aureus* lineages co-existed with *E. helvum*. The Straw-Coloured Fruit Bat in Ile-Ife, Nigeria is colonized predominantly by ST1725, ST1726, ST2463 and ST2470 with distinct genotypic characteristics that are rarely found in humans. This study has demonstrated on the possible existence of a reservoir of indigenous and anciently-divergent *S. aureus* clones among mammals in Africa.

## Background

Bats (Order: *Chiroptera*) are the only mammals capable of true sustainable flight and one of the most diverse and species rich mammals on earth [1]. They assist in the regulation of insect populations in their habitats, pollination of flowers and dispersal of seeds of economically important tress, and these ecological roles support forest regeneration and maintenance [2]. However, they roost near human habitation and their association with emerging infections has increased attention on these flying mammals as vectors of zoonotic pathogens [3-5]. The bat species *Eidolon helvum* is grouped under the suborder *Megachiroptera*, and it is the most widely distributed Straw-Coloured Fruit Bat which is found in the forest and savannah zones of sub-Saharan Africa [6,7]. The prime habitats for *E. helvum* are the tropical forest and typically roost in colonies on tall trees like *Eucalyptus saligna* and *Cocos nucifera*[8].

*Staphylococcus aureus* is part of the normal flora of the skin and mucous membrane of a wide variety of mammals and birds, and recent studies have indicated that animals could be a source of *S. aureus* infections in humans [9-11]. The main campus of the Obafemi Awolowo University, Ile-Ife (OAU) Nigeria, is colonized by a large population of *E. helvum*[12,13], but faecal contamination and pollution of the environment by these migratory mammals is a problem, moreover, the public health implications of their activities are not known. This study characterized *S. aureus* obtained from faecal samples of bats that colonize the main campus of the institution, with a view to understanding the clonal nature and diversity of the isolates, and to determine the possible risk of dissemination of *S. aureus* from bats to humans in the community through faecal shedding.

## Results and Discussion

**Figure 1:**
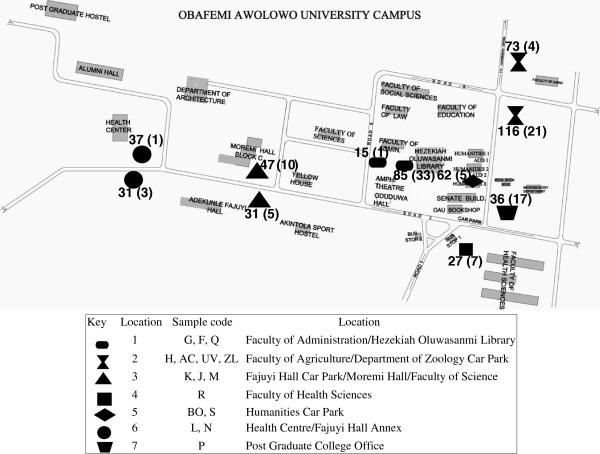
**Map of Obafemi Awolowo University (OAU) campus showing the sampling site/roosting habitat of the Straw-Coloured Fruit Bat (*E. helvum*).** The number of samples (in each site) and *S. aureus* isolates (in parenthesis) are indicated.

A total of 107 *S. aureus* isolates were obtained from 560 faecal samples of *E. helvum* based on phenotypic identification. Moreover, they were all genotypically confirmed by *hsp60* partial sequencing, and there was excellent agreement between the phenotypic and molecular methods in the identification of the isolates. The number of samples and *S. aureus* isolates in each sampling site are indicated in Figure 1. Antibiotic susceptibility testing is paramount for monitoring resistance in commensal bacteria and various pathogens of clinical importance. In this study, all the isolates were susceptible to oxacillin, cefoxitin, tetracycline, chloramphenicol, gentamicin and mupirocin. However, four (3.7%) isolates were resistant to penicillin, while six (5.6%) and eight (7.4%) isolates were resistant to ciprofloxacin and erythromycin, respectively. None of the isolates exhibited inducible resistance however, 3.7% were constitutively resistant to clindamycin (Table 1). Studies have reported faecal carriage of methicillin-resistant *S. aureus* (MRSA) in animals [14,15]. However, MRSA was not detected in this study which is similar to recent reports on analysis of faecal samples from swine and feedlot cattle [16,17]. The low rate of resistance to different classes of antibiotics observed among the isolates in this study suggests that these migratory mammals may not have been exposed to the selective pressure of antimicrobial agents.

**Table 1:** **Antibiotic susceptibility of 107*S. aureus*isolates from faecal samples of*E. helvum*in Nigeria**

**Antibiotics (disk content in μg)**	**Number of isolates**		**Resistance rate (%)**
	**S**	**R**	
Penicillin (10 units)	103	4	3.7%
Oxacillin (1 μg)	107	0	0%
Cefoxitin (30 μg)	107	0	0%
Erythromycin (15 μg)	99	8	7.5%
Clindamycin (2 μg)	103	4	3.7%
Tetracycline (30 μg)	107	0	0%
Ciprofloxacin (5 μg)	101	6	5.6%
Chloramphenicol (30 μg)	107	0	0%
Fusidic Acid (10 μg)	104	3	2.8%
Gentamicin (10 μg)	107	0	0%
Mupirocin (5 μg and 200 μg)	107	0	0%

*S*= Susceptible; *R*= Resistant.

Molecular typing has been useful in understanding the epidemiology of *S. aureus* from animal and human hosts [18]. *S. aureus* is highly clonal in nature and though some are exclusively adapted to specific hosts [19], others are able to colonize multiple hosts [20-22]. Of the 107 *S. aureus* isolates, 70 (representing isolates obtained from faecal samples in the various sites) were randomly selected and further characterized. All the isolates were PVL-negative and 65 (92.9%) were grouped with coagulase (*coa*) type VI, but 5 (7.1%) were non-typeable. The accessory gene regulator (a*gr*) typing classified 69 of the 70 isolates into the following: type I (12; 17.1%), type II (3; 4.3%), type III (1; 1.4%) and type IV (53; 75.7%). Based on their genotypic characteristics, ten representative isolates were selected for MLST and nine new sequence types: ST1725, ST1726, ST1727, ST2463-ST2467 and ST2470 were identified, and the sequences for the housekeeping genes have been deposited in the MLST database (http://www.mlst.net), while one representative isolate (Q22) was assigned with ST15. Overall, the 70 isolates were assigned into five main genotypes A to E (Table 2).

**Table 2:** **Genotypes identified in 70*S. aureus*isolates from faecal samples of*E. helvum*in Nigeria**

***hsp60*allelic type**	***coa***	***agr***	**Representative isolate ID**	**Allele**	**No of isolates (%)**
				***arcC, aroE, glpf, gmk, pta, tpi, yqiL***	
				**MLST (ST)**	
A0	VI	IV	F10	1-13-84-1-12-5-11 (ST1725)	14 (20)
A1	VI	IV			02 (2.9)
B0	VI	IV	AC19	1-13-84-1-184-5-11 (ST1726)	21 (30)
B1	VI	IV			01 (1.4)
B2	VI	NT	R5	193-245-227-136-185-5-11 (ST1727)	01 (1.4)
C0	VI	IV	AC10	211-303-303-142-195-211-274 (ST2463)	15 (21.4)
C1	NT	I	F9	270-305-248-188-266-202-186 (ST2464)	01 (1.4)
C2	NT	II	P1	211-305-248-188-195-202-275 (ST2465)	01 (1.4)
C3	NT	II	Q15	270-307-304-143-195-202-276 (ST2466)	01 (1.4)
C4	NT	III	R3	271-356-248-189-267-202-186 (ST2467)	01 (1.4)
D0	VI	I			09 (12.9)
D1	VI	I	F16	272-357-306-190-268-270-277 (ST2470)	01 (1.4)
D2	VI	I			01 (1.4)
E0	NT	II	Q22	13-13-1-1-12-11-13 (ST15)	01 (1.4)
			TOTAL		70 (100)

*NT*: Non-typeable.

*coa*: coagulase gene.

*agr*: accessory gene regulator.

All the isolates were PVL negative.

**Figure 2:**
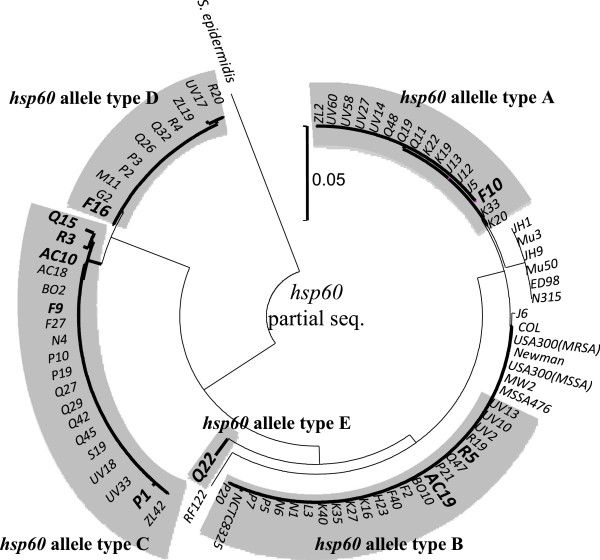
**Phylogenetic tree based on *hsp60 *partial sequences of 70 *S. aureus *isolates from *E. helvum.*** This tree was constructed by the neighbor-joining method, using MEGA ver. 5.05.

**Figure 3:**
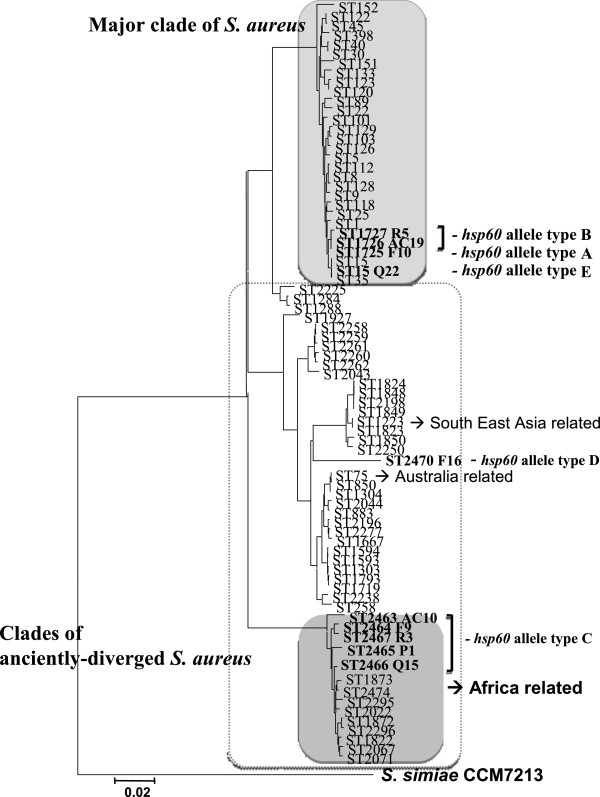
**Phylogenetic tree based on concatenated *arcC, aroE, glpF, gmk, pta, tpi *and *yqiL *sequences of representative *S. aureus *isolates (F10, AC19, R5, AC10, F9, P1, Q15, R3, F16 and Q22).** This tree was constructed by the neighbor-joining method, using MEGA ver. 5.05.

As shown in Figure 2, there was a clear phylogenetic out-group among the *S. aureus* taxon consisting of isolates in the *hsp60*-allele types C and D, which suggests that these genotypes diverged long before clones belonging to the major *S. aureus* clades exhibited the current size of genetic divergence. Moreover, based on concatenated sequences of seven genes used in MLST, isolates in *hsp60*-allele type C were closely related with *S. aureus* ST1822 and associated clones, and type D isolates with ST75, ST883 and ST1223 (Figure 3). We have tentatively designated these isolates as anciently-diverged *S. aureus*. Some studies had previously reported that divergent *S. aureus* ST75 (*agr* type I) and ST883 (*agr* type IV) originated in northern Australia, while ST1223-related clones were found in South East Asia [23-25]. Moreover, *S. aureus* isolates assigned with ST1822-related clones have been identified in African monkeys [26]. In this study, we identified divergent clones (ST2463-ST2467, ST2470) among Straw-Coloured Fruit Bats in Nigeria, which suggests that anciently-diverged *S. aureus* have not only been distributed in Australia and South East Asia, but also among mammals in Africa. These lineages evolved independently from major *S. aureus* populations over an extended period of time, and may be a new subspecies of *S. aureus*. A recent study had reported that chromosomal recombination had occurred at *coa* and *agr* loci at a uniform rate [27]. Therefore, it is difficult to identify the prototype of these genes. The *agr* type I or IV and the *coa* type VI, which were found most frequently in the anciently-diverged *S. aureus* isolates, may be the closest relation to the origin of *agr* and *coa* genes, respectively.

## Conclusions

This study isolated *S. aureus* from faecal samples of *E. helvum*, a migratory mammal with an abundant population in OAU, Ile-Ife, Nigeria, and represents the first molecular study on *S. aureus* colonization of bats in Africa. The isolates were largely susceptible to a number of antibiotics. The combination of coagulase gene type VI and *agr* type IV are rare among *S. aureus* isolates associated with humans [28-31], and the evidence that isolates in group C were closely related with divergent ST1822-related clones identified in African monkeys, and group D isolates with ST75, ST883 and ST1223 indicate that there is the possible existence of a reservoir of indigenous and anciently-diverged clones among mammals in Africa.

## Methods

### Sample sites

A total of eleven roosting sites located in the academic area and the students’ hostel in OAU, Ile-Ife were identified for the study (Figure 1), and the duration for sample collection was from January 2008 to September 2008, February to May 2009, and February 2010. The faecal samples were obtained once a month in a designated sampling site between 6-7am by a non-invasive method in which three sterilized piece (36 × 45 inches) of cotton material were spread under the roosting trees. Fresh faecal samples were collected with sterile swab sticks and conveyed promptly to the Department of Microbiology Laboratory (OAU) for microbiological analysis.

### Isolation and identification of *S. aureus* isolates

The swab stick was inserted into a test tube containing 3 ml of sterile nutrient broth (Biolab, supplied by Merck, Johannesburg, South Africa), swirled briefly to discharge the contents into the medium, and the culture was incubated at 37°C overnight. Thereafter, a loopful was streaked on mannitol salt agar (MSA) (Biolab, supplied by Merck, Johannesburg, South Africa) and incubated at 37°C for 48 hours. Preliminary identification of *S. aureus* was based on positive Gram stain, and positive results for catalase, coagulase (tube method) and DNase tests. The procedure described previously [32] was employed for DNA isolation. In summary, a single colony was suspended to a McFarland 1.0 standard in 100 μl of TE buffer (10 mM Tris, 1 mM EDTA, pH 8.0) with 10 U of achromopeptidase (Wako Chemical, Co. Ltd.), and the suspension was incubated at 55°C for 10 min. The supernatant was used as crude DNA for PCR. Molecular identification and confirmation of the isolates was based on sequencing analysis of the *hsp60* gene as previously reported [33]. PCR products were sequenced by using a Big Dye Terminator (version 3.1) cycle sequencing kit (Applied Biosystems, Foster City, CA) with an ABI Prism 3100 genetic analyzer (Applied Biosystems).

### Antibiotic susceptibility testing

The susceptibility testing of the isolates to 11 antibiotics was performed using the disk diffusion method and the following antibiotics were tested: penicillin (10 units), oxacillin (1 μg), cefoxitin (30 μg), erythromycin (15 μg), clindamycin (2 μg), tetracycline (30 μg), ciprofloxacin (5 μg), chloramphenicol (30 μg), fusidic acid (10 μg) gentamicin (10 μg) and mupirocin (5 μg and 200 μg). *S. aureus* ATCC 25923 was the control strain for the susceptibility testing. The result was interpreted as resistant or susceptible based on the interpretative standard according to the Clinical Laboratory Standards Institute (CLSI) manual for bacterial isolates from animals [34]. Interpretative zone diameter for resistance and susceptibility breakpoints to fusidic acid and mupirocin which are not stated in the CLSI guidelines were considered as described previously [35,36]. The D-test for determining inducible resistance of clindamycin using erythromycin was performed. A truncated or blunted clindamycin zone of inhibition (D-Shape) indicated inducible resistance. Constitutive resistance was recognized by a clindamycin zone diameter of ≤14 mm [37].

### Molecular characterization of the *S. aureus* isolates

Characterization of 70 isolates was determined by detection of the Panton Valentine Leukocidin (PVL) gene [38], *agr*[39] and *coa* gene typing [40]. The MAFFT program was used for multiple alignment of the *hsp60* partial sequences, and a phylogenetic tree was constructed by the neighbor-joining and bootstrap methods, using MEGA ver. 5.05 [41]. Furthermore, MLST [42] was carried out on representative *S. aureus* isolates (based on *hsp60* allelic type, coagulase and *agr* typing). The amplified PCR products were sequenced, and STs were determined for each isolate based on the alleles identified at each of the seven loci using the *S. aureus* MLST database (http://www.mlst.net). For six representative isolates (AC10, F9, P1, F16, Q15 and R13), we were unable to amplify the *aroE* and or *glpF* genes using the standard MLST primers. Therefore degenerate primers CC75dege-aroE-F (5’-WTGCAGTWATHGGWRRYCC-3’), CC75dege-aroE-R (5’-GGWWTATAAAYAATRT CACT-3’), CC75aroEseq-F (5’-CCAATTGAGCATTCYTTATC-3’), CC75dege-glpF-F (5’-GCWGAATTYHT DGGWACWGC-3’), CC75dege-glpF-R (5’-ATWGGYA AWATHGCATGWGC’), and CC75glpF-seq-R (5’-GCAT GTGCAATTCTTGGDC’), were designed by multiple alignment of amino acid sequences of each gene with complete genomes of *S. aureus*, *S. epidermidis*, *S. haemolyticus* and *S. lugdunensis* from the KEGG database (http://www.genome.jp/kegg/). Sequences of *arcC, aroE, glpf, gmk, pta, tpi* and *yqiL* in *S. simiae*, which was used as an outgroup, were obtained from the draft genome sequence of *S. simiae* CCM7213 [43]. A phylogenetic tree was constructed based on concatenated *arcC, aroE, glpF, gmk, pta, tpi* and *yqiL* sequences using the neighbor-joining method, using MEGA ver. 5.05.

## Abbreviations

OAU: Obafemi Awolowo University; PVL: Panton Valentine Leukocidin; Agr: Accessory gene regulator; Coag: Coagulase; MLST: Multilocus sequence typing; ST: Sequence type; *E. helvum*: *Eidolon helvum*; *S. aureus*: *Staphylococcus aureus*; MSA: Mannitol salt agar; DNase: Deoxyribonuclease; CLSI: Clinical Laboratory Standards Institute; MRSA: Methicillin resistant *Staphylococcus aureus*.

## Competing interests

The authors declare that they have no competing interest.

## Authors’ contributions

AS, OA, TS conceived the study, BA conducted the sample collection, preliminary identification and susceptibility testing of the isolates; TS carried out the molecular characterization. All authors read and approved the final version of the manuscript.
